# Nailfold Videocapillaroscopy for the Evaluation of Peripheral Microangiopathy in Rheumatoid Arthritis

**DOI:** 10.3390/life12081167

**Published:** 2022-07-31

**Authors:** Panagiota Anyfanti, Elena Angeloudi, Athanasia Dara, Alexandra Arvanitaki, Eleni Bekiari, George D. Kitas, Theodoros Dimitroulas

**Affiliations:** 1 Second Medical Department, Hippokration Hospital, Aristotle University of Thessaloniki, 54642 Thessaloniki, Greece; elena-angeloudi@hotmail.com (E.A.); bekiarie@auth.gr (E.B.); 2 Fourth Department of Internal Medicine, Hippokration Hospital, Aristotle University of Thessaloniki, 54642 Thessaloniki, Greece; athadara@auth.gr (A.D.); alexandra.arvanit@gmail.com (A.A.); dimitrou@auth.gr (T.D.); 3 First Department of Cardiology, AHEPA University Hospital, Medical School, Aristotle University of Thessaloniki, 54621 Thessaloniki, Greece; 4 Department of Rheumatology, Russells Hall Hospital, Dudley Group NHS Foundation Trust, Dudley DY1 2HQ, UK; george.kitas@nhs.net; 5 School of Sport, Exercise and Rehabilitation Sciences, University of Birmingham, Birmingham B15 2TT, UK

**Keywords:** rheumatoid arthritis, nailfold videocapillaroscopy, microcirculation

## Abstract

Rheumatoid arthritis (RA) is a chronic and refractory autoimmune joint disease that affects multiple organs. Several methods have been applied for the study of microvascular endothelial dysfunction, which is considered an important component of vascular disease in RA. Implementation of nailfold videocapillaroscopy (NVC) represents a viable choice, as the skin is an easily accessible window for the non-invasive, real-time assessment of subtle microcirculation abnormalities. Although NVC is routinely used in the rheumatology field, especially for the diagnostic workout of Raynaud’s phenomenon, accumulating evidence suggests a role in the evaluation of systemic vasculopathy associated with autoimmune rheumatic disorders. The current paper aims to provide an overview of NVC as a valuable clinical aid for the assessment of peripheral microcirculation in RA. Previous studies characterizing the capillaroscopic pattern in RA are summarized, along with associations with disease-related characteristics. Most available reports have mainly focused on the descriptions of non-specific morphological alterations that may reflect endothelial injury over the course of the disease. Still, the exact pattern of structural and functional capillaroscopic alterations and their clinical significance in RA remains a subject of ongoing research.

## 1. Introduction 

Rheumatoid arthritis (RA) is the most prevalent autoimmune inflammatory rheumatic disease [1], characterized by varying levels of physical disability and chronic pain. RA is accompanied by multiple extra-articular manifestations and comorbidities. Cardiovascular disease is one of the most common and preventable comorbidities and one of the leading causes of death, the burden of which is increased by as much as 50% compared to the general population among patients with RA [2]. RA has been acknowledged as a non-conventional cardiovascular disease risk factor [3] associated with accelerated atherosclerosis and microvascular endothelial dysfunction resulting in vascular damage [4,5]. The latter is considered an extra-articular manifestation of RA secondary to complex interactions between chronic inflammation and autoimmune dysregulation in combination with the increased prevalence of traditional cardiovascular disease risk factors [6,7]. 

Pulmonary disease, most often in the form of primary and secondary interstitial lung disease, occurs in 30–40% of RA patients and represents the second leading cause of death in patients with RA [6]. Other common and potentially preventable comorbidities include gastrointestinal disease, in particular liver dysfunction related directly to RA or ongoing therapies, renal dysfunction (demonstrated as glomerulonephritis, drug-induced nephropathy, or amyloidosis), and neurological disease [7]. Other than rheumatoid nodules, rheumatoid vasculitis is a frequent extra-articular manifestation that typically causes severe injury to the affected blood vessels (most frequently small vessels), involves different organs (including the heart, lungs, and kidneys), and aggravates RA-related morbidity and mortality [8]. In light of the consistently high rates of morbidity and mortality, effective health care strategies are urgently warranted to identify high-risk individuals with RA at an early stage, before the establishment of detrimental disease-related manifestations. 

Although traditionally used for several decades as a bedside aid in rheumatology for the diagnosis and monitoring of peripheral vasculopathy in connective tissue diseases [9], nailfold videocapillaroscopy (NVC) has recently emerged as a non-invasive, inexpensive, easily applicable technique for the study of peripheral microangiopathy. It is reasonable that direct visualization of human microcirculation could provide valuable information regarding vascular health at a preclinical level. Under this prism, nailfold capillaroscopic examination might emerge as an instrumental modality with a complementary role in the early detection of microvascular pathology associated with RA and its extra-articular manifestations. However, several gaps in knowledge remain regarding the evaluation of microangiopathy with NVC in RA, especially with regard to specific capillaroscopic patterns and their clinical significance. In the present review paper, we aim to provide an overview of the data supporting the role of NVC in the evaluation of peripheral microangiopathy in RA. A brief description of the method is provided and data favoring the association of peripheral dermal capillary alterations with systemic microangiopathy are discussed. Special emphasis is placed on capillaroscopic findings encountered in patients with RA and their potential association with disease-related manifestations and characteristics. To this end, a PubMed search was performed to identify relevant articles using the following medical terms: “rheumatoid arthritis”; “capillaroscopy”; “nailfold capillaroscopy”, and “nailfold videocapillaroscopy”. 

## 2. Nailfold Capillaroscopy: Description of the Method

Nailfold capillaroscopy is a non-invasive imaging technique applied to an in vivo, dynamic, two-dimensional projection of the three-dimensional capillary network of the studied organ, typically the skin [10]. NVC is applied to the finger nailfold to produce images of dermal microcirculation, which corresponds to the terminal vascular network of the systemic circulation [11]. Capillaries are the thinnest component, formed by only two layers of cells—an inner layer of endothelial cells and an outer layer of epithelial cells—and shaped into an afferent (arterial) limb, an efferent (venous) limb, and a transitional zone [12]. Nailfold capillaries were first detected in the 17th century using primeval magnifying equipment. In the 19th century, the first link between capillary abnormalities and certain medical entities was introduced based on the innovative research work of Maurice Raynaud [13]. In the 21st century, the results of numerous publications established its value as a routine clinical procedure in rheumatology, especially among patients with systemic sclerosis for whom it plays a substantial role in terms of diagnosis, prognosis, and monitoring, as described in detail elsewhere [9,10]. In addition, NVC is of outstanding importance in the differentiation between primary and secondary Raynaud’s phenomenon, and the monitoring of patients with Raynaud’s phenomenon as their only presenting symptom [14].

### 2.1. Description of NVC Technique

NVC traditionally assesses skin microcirculation within the nailfold, where the major axis of the capillaries runs parallel to the skin surface, allowing for a detailed evaluation of their morphology [15]. During the examination, the patient is in a sitting position and the hands are placed at the level of the heart. To prevent vasospasm, the patient’s acclimatization to a temperature-controlled room (20–22 °C) is required [16]. Thumbs and fingers affected by either infection or recent trauma are not analyzed [17]. A drop of immersion oil is applied prior to the procedure to improve the resolution. 

Nailfold capillaroscopy can be performed using either a low-magnification lens (×20) or a high-magnification lens (×200). Low-magnification lenses offer a panoramic view of the whole microvascular network. On the other hand, a videocapillaroscope not only allows for low magnification but also has the potential of providing sequential high magnifications using developed computerized systems, which enable detailed observations of separate capillaries and can subsequently analyze each capillary at different time points. Another advantage of this tool is that it consists of a probe that is moved to the finger of the patient and allows for direct contact with the nailfold, thus facilitating the study of patients with severe joint contractions [18]. Practically, NVC uses optical videocapillaroscopy equipped with a ×200 magnification probe and is simultaneously connected to image analysis software. Hence, images can be stored in a digital form, providing the potential to use them for objective comparisons among subsequent measurements. NVC may also detect blood flow at the level of the microvessels and provide information on the functional state of the microvasculature [19].

### 2.2. Capillaroscopic Microvascular Parameters Detected with NVC

During NVC examination, the capillaries are quantitatively and qualitatively assessed. In a qualitative assessment, an overall interpretation is acquired after documenting the visibility of the image regarding the morphology, density, and dimensions of the capillaries, as well as their architecture. A qualitative assessment is especially useful in screening for systemic sclerosis in patients with Raynaud’s phenomenon based on the distinction between normal and abnormal capillaroscopic morphology [18]. A quantitative or (semi)-quantitative evaluation is based on manual automated measurements of certain parameters of each capillary per linear millimeter, including the dimensions and density. Semi-quantitation gives a score between 0 and 3 per capillaroscopic characteristic and is applied in associative and prediction investigations [18]. The main qualitative and quantitative morphological characteristics observed and documented are capillary architecture and organization; capillary morphology; capillary density (number of capillary loops per millimeter); capillary size (including enlarged and giant capillaries); microhemorrhages; avascular areas and neoangiogenesis [20,21]. 

### 2.3. Normal and Altered Capillaroscopic Patterns

In general, a preserved microvascular function is characterized by a clear visualization of architecture, that is, the absence of bleeding and exudates, as well as a preserved perfusion with an uninterrupted blood flow across the capillaries. Nailfold capillaries usually demonstrate a regular architecture, uniform shape, distribution, and diameter, and most of them show a hairpin or U-shape appearance [22]. Their limbs are parallel to each other, without crossing over or overlapping [23]. The venous limb is often greater in diameter than the arterial limb, but the venous limb/arterial limb diameter ratio does not exceed 2:1 [21]. The subpapillary venous plexus can be observed in 10–30% of healthy persons [24]. In healthy subjects, giant capillaries and avascular areas are typically absent. However, the presence of isolated abnormalities does not necessarily indicate disease [25]. A pilot study by the EULAR Study Group on Microcirculation in Rheumatic Diseases proposed a simple definition to describe the morphology of single capillaries as being normal or abnormal. The simple definitions proposed were: Definition 0—normal or non-specific (defined as hairpin, crossing, or tortuous); Definition 1—abnormal (not hairpin, not tortuous, and not crossing); Definition 2—not evaluable (whenever the rater is undecided in classifying between normal and abnormal) [26]. Although moderate reliability of this definition was obtained by raters in this study, a subsequent multicenter international study conducted during the seventh EULAR course on capillaroscopy demonstrated the excellent reliability of the optimized simple capillaroscopic definition of normal and abnormal morphologies of capillaries, even when used by rheumatologists with varying levels of expertise in capillaroscopy [27].

Abnormal alterations of capillary morphology stand in contrast to the physiologic capillary pattern. Capillary abnormalities are variable regarding certain morphological or structural characteristics, such as the size and density of capillaries [28]. Due to increasing interest in NVC, there have been considerable efforts to develop a uniform nomenclature. The most important capillary alterations and their clinical significance have been described in detail elsewhere [17,29,30]. 

## 3. Abnormal NVC Findings in Patients with RA 

Although NVC has emerged as a valuable tool for the evaluation of systemic microangiopathy in autoimmune connective tissue diseases, qualitative and quantitative capillaroscopic alterations in RA remain less well-defined. In general, the presence of a specific capillaroscopic pattern in RA patients has been a subject of investigation over several decades. Studies describing morphological findings in RA with the use of NVC are summarized in Table 1. 

### 3.1. Morphological Abnormalities

One of the oldest studies by Redisch et al. included 31 patients with RA, and although a scleroderma pattern was not observed, elongated capillaries, increased tortuosity, and prominent subpapillary plexus were the most prominent abnormal capillaroscopic findings [31]. In 1995, Altomonte et al. published their results in a cohort of 32 RA patients in whom elongated and tortuous capillaries were likewise the main though non-specific abnormalities [32]. Other non-specific capillaroscopic findings described in some of the oldest studies in the field vary from minor non-specific abnormalities (very thin capillary loops) [33] to erythrocyte aggregation, capillary neoangiogenesis, and cutaneous atrophy in the majority of cases [34]. Interestingly, the prevalence of capillaroscopic abnormalities has varied significantly among the oldest studies [33,34] reaching 93.8% of patients with RA [35], although the absence of a control group limits the generalizability of these results.

Further exploring this study field, Lambova et al. [36] demonstrated that the most frequent findings during nailfold capillaroscopic examination of patients with RA were prominent subpapillary plexus in 69% and elongated capillaries in 58%. The mean capillary length in RA patients was significantly longer than those in healthy individuals. Moreover, the diameters of the arterial and the venous limb of the capillary loops in patients with RA and secondary Raynaud’s phenomenon were found to be significantly wider as compared to those without Raynaud’s phenomenon. Additionally, dilated capillaries were found not only in RA patients with Raynaud’s phenomenon but also in patients without clinical signs consistent with peripheral vasospasm. Of note is the fact that compared to healthy controls, the diameters of the capillary loops of RA patients without secondary Raynaud’s phenomenon were also significantly wider, a finding that may be partially explained by endothelial damage in different mechanisms during the course of the disease in these patients. This study reported for the first time the presence of a scleroderma-like pattern in RA, which was subsequently confirmed by other authors, as analyzed below. In this study, a low incidence (14.5%) of a scleroderma-like pattern was observed, and secondary Raynaud’s phenomenon was present in all RA patients with a scleroderma-like capillaroscopic pattern [36]. 

A recent study examined a large cohort of 201 RA patients in comparison with 50 healthy controls. Non-specific capillaroscopic findings were observed in almost half (45.77%) of the RA patients, who presented an increased prevalence of tortuosity and dilated and bushy capillaries. Consistent with previous reports, nonspecific capillaroscopic findings were more frequent among RA patients with Raynaud’s phenomenon [37]. Rather surprisingly, another study of 430 RA patients reported normal capillaroscopic patterns only in a small minority of patients (7.2%), scleroderma patterns in 20.9%, and nonspecific abnormalities in the majority of patients (71.8%). Tortuosity and angiogenesis were present in the vast majority of patients with RA (99.5% and 74.7%, respectively) [38]. Likewise, tortuosity was evident in the whole sample (100%) of RA patients who were examined by Bernardino et al. as a subset of a large cohort of patients with rheumatic diseases. A large prevalence of crossing capillaries (100%), enlarged capillaries (68.2%), and hemorrhages (59.1%) was also found, whereas neoangiogenesis only affected 4.55% [39]. The subjective assessment of such changes from different operators might largely account for the discrepancies in the reported prevalence of different NVC abnormalities. Overall, the most commonly described capillaroscopic patterns in RA appear to be largely non-specific [1,2].

### 3.2. Functional Abnormalities

While morphological capillaroscopic features in RA have been addressed in previous reports, fewer studies have focused on functional abnormalities. In an older study, functional impairment of the capillary wall was detected after the intravenous injection of fluorescein sodium using dynamic fluorescence nailfold NVC in patients with RA [40]. Other microvascular alterations in normal bloodstream have been described in RA patients, including reduced reactive hyperemia in cutaneous capillaries compared to patients with osteoarthritis [41]. Reduced capillary blood flow and cold-induced vasospasm have been additionally observed using nailfold NVC combined with a cold provocation test [42]. 

## 4. Association of Abnormal NVC Findings with Other Disease Features in RA

NVC has been widely applied in the field of rheumatic diseases, especially in systemic sclerosis with indisputable clinical value in diagnostic terms, as well as the assessment and follow-up of peripheral digital vasculopathy. Peripheral microvascular changes in systemic sclerosis detected with NVC correlate with diffuse vascular impairment (e.g., myocardial dysfunction, pulmonary vascular disease) and reflect the severity of visceral organ involvement [43]. For instance, the extent of peripheral microvasculopathy assessed by NVC has been found to correlate with the presence of pulmonary arterial hypertension in a recent meta-analysis of patients with systemic sclerosis [44]. Importantly, worsening NVC patterns of microvasculopathy in systemic sclerosis strongly correlate with higher estimated CVD risk [11], as well as independent markers of endothelial dysfunction, such as arterial stiffness [11,45] and increased levels of uric acid [46]. Last but not least, decreased capillary density and increased loop width have been described in patients with pulmonary arterial hypertension [47] due to congenital heart disease [48], various autoimmune diseases [16], chronic thromboembolic pulmonary hypertension [49], and essential hypertension, in whom it is considered to reflect the maladaptive cardiovascular responses as part of generalized microangiopathy [50]. However, the association of NVC findings with signs of generalized vasculopathy remains less well-established in RA. A summary of the clinical and laboratory findings (including auto-immune parameters) in association with capillaroscopic findings in RA is presented in Table 1.

### 4.1. Correlation with Clinical Parameters

In most studies, NVC findings have not been associated with the disease activity [37,39,51,52], duration [32,52], or functional activity class of RA [32]. One study demonstrated a weak relationship between tortuosity and the duration of disease [37]. Longer disease duration, but not morning stiffness or the number of tender or swollen joints, was associated with the presence of severe capillaroscopic abnormalities in one of the oldest studies by Kuryliszyn-Moskal et al. The same study provided evidence that NVC findings are indicative of systemic involvement in RA. In particular, patients with severe capillary abnormalities showed a significantly higher frequency of joint erosions and extra-articular involvement compared to those with mild and moderate vascular changes. Skin manifestations as part of cutaneous vasculitis were more common in patients with severe vascular changes compared to those with mild or moderate capillary abnormalities. Furthermore, all RA patients with systemic vasculitis showed moderate or severe microvascular abnormalities, while severe capillaroscopic changes were found only in 12.8% (6 or 47) of patients without systemic involvement [35]. The same group had previously shown that all patients with extra-articular manifestations of RA showed moderate or severe vascular changes in nailfold capillaroscopy compared to 61% of RA patients without clinical signs of vasculitis who also demonstrated moderate-to-severe capillaroscopic abnormalities [53]. 

Consistent with the above, Witkowska et al. demonstrated that moderate to severe changes in nailfold capillaroscopy were found in all patients with extra-articular manifestations [54]. These results generated the hypothesis that abnormal microvascular findings in RA may also reflect digital vasculitis and systemic organ involvement in the context of systemic vasculitis, which is a well-known extra-articular feature of RA [55]. However, this hypothesis needs to be addressed with circumspection, as tortuous and crossing capillaries, which are frequently described as abnormal NVC findings in RA, are common findings in healthy subjects and do not actually represent a feature of microangiopathy [56]. Moreover, functional impairment of the capillary wall has been detected in patients with RA regardless of the concomitant vasculitis [40].

On the contrary, neoangiogenesis indicates microangiopathy especially when it occurs in association with low capillary density, avascular areas, and capillary derangement, and these capillaroscopic features should be carefully addressed in light of their clinical significance [57]. A scleroderma-like pattern is not necessarily associated with an overlap syndrome, as was previously clearly defined [58]. However, these capillaroscopic changes can be found in RA patients with peripheral digital vasculitis and in patients with secondary Raynaud’s phenomenon [36,37,56]. Lambova et al. observed a scleroderma-like pattern in 9/62 (14.5%) of the included RA patients. In all RA patients with scleroderma-like capillaroscopic pattern (9/9), secondary RP was present, 2/9 presented secondary vasculitis, and 1/9 had an overlap syndrome with systemic lupus erythematosus, secondary RP, and secondary vasculitis [36]. Despite relevant data in patients with systemic sclerosis, as mentioned earlier, whether and to what extent a scleroderma-like pattern in RA correlates with visceral organ involvement remains less well-established. In a large cohort of 759 patients with Raynaud’s phenomenon diagnosed with different rheumatic diseases, a scleroderma-like pattern was associated with a higher prevalence of abnormal pulmonary function tests, independently of the presence of an systemic sclerosis diagnosis. However, only 15 individuals were patients with a definite diagnosis of RA, of whom only 13% presented a scleroderma pattern in NVC [59]. Still, the presence of such changes in patients with RA requires closer monitoring and regular follow-up for the possibility of the development of systemic rheumatic disease different from inflammatory arthritis. Finally, the association of NVC parameters with atherosclerotic markers in RA remains under investigation. This could be particularly important in light of the increased cardiovascular burden associated with RA, as preliminary data have shown that capillary abnormalities, specifically capillary rarefaction, correlates with cardiovascular disease risk.

### 4.2. Correlation with Laboratory Parameters

Previous studies on RA have failed to reveal significant associations between NVC findings and a rheumatoid factor [32,37] or anti-cyclic citrullinated peptide positivity [37]. Higher subpapillary venous plexus visibility has been evidenced in patients presenting antinuclear antibodies and antibodies against rheumatoid-arthritis-associated nuclear antigen (anti-RANA) antibodies, possibly as an expression of the endothelial damage induced by antinuclear antibodies in vessel walls [32]. Capillary rarefaction has been associated with serum levels of C-reactive protein in patients with RA [52]. One study reported significantly higher soluble CD4 (sCD4) in RA patients with severe vasculitis compared to the group with mild vascular changes, with no differences in serum levels of tumor necrosis factor-alpha (TNF-α), interleukin (IL)-6, and soluble IL-6 receptor (sIL-6R) among groups with a different capillaroscopic score [35]. The same group failed to demonstrate a significant association between soluble intercellular adhesion molecule-1 (sICAM-1) levels and capillaroscopic findings [53], although a higher sICAM-1 level seemed to reflect more intensive vascular changes in capillaroscopy in a subsequent study [54].

## 5. Future Perspectives

Although the dermal capillary network represents an “open window” for the study of microcirculation, prospective data evaluating the evolution of NVC findings throughout time in patients with RA is currently missing, and several questions remain unanswered. A crucial point is the potential predictive value of NVC abnormalities detected as part of undifferentiated or early arthritis for the subsequent diagnosis of RA. In a prospective study of 250 children and adolescents with Raynaud’s phenomenon who were followed up every 6 months for 1 to 6 years after the first capillaroscopy, nonspecific capillary changes occurred in 3 out of 10 (30%) patients who were subsequently diagnosed with RA, but these were not predictive of the future development of RA or juvenile-onset RA [60]. Another developing field of research is the potential association of NVC findings with markers of atherosclerosis, which suggests a role of NVC in the cardiovascular risk stratification of patients with RA. Finally, it remains to be clarified whether and which capillaroscopic patterns in RA may improve through appropriate interventions, and if so, whether the remission of such signs might be accompanied by improved extra-articular manifestations, systemic organ involvement, and overall disease prognosis. Long-term, appropriately designed prospective studies are urgently needed to address the knowledge gaps and establish the clinical value of NVC in RA.

## 6. Conclusions 

Accumulating data support the role of NVC in the assessment of peripheral microangiopathy associated with systemic diseases. NVC provides direct visualization of dermal capillary morphology and structure, along with a real-time assessment of their functional status and hemorheological dynamics. Although not a novel method in the rheumatology field, most available studies with NVC in RA solely provide a description of non-specific morphological abnormalities, most commonly elongated capillaries, increased tortuosity, and prominent subpapillary plexus. Such alterations are presumed to reflect endothelial injury due to systemic inflammation over the course of the disease. Far fewer studies have used NVC to evaluate the functional status of dermal capillaries in RA or applied a quantitative approach. There is a need for long-term, wide-scale follow-up studies to specify whether and which capillaroscopic patterns can be correlated with clinical and laboratory findings in RA, and predict not only its clinical course but also the development of extra-articular manifestations and disease-related comorbidities.

## Figures and Tables

**Table 1:** Morphological alterations detected by nailfold videocapillaroscopy in RA.

Study, Year of Publication	Type of Study	Study Population	Main Patterns in Patients with RA	Reported Associations of NFC Findings with Other Disease Features
Redisch et al., 1970	Case-control study	31 RA patients, 80 patients with other rheumatic diseases, and 200 healthy subjects	Paucity of visible capillaries (71%); increased tortuosity (48%); linear elongation of loops (42%); venular plexus widening and engorgement (42%); widening of all three limbs (42%); apical widening (16%); disarrangement of capillary polarity (13%); focal narrowing and widening of efferent limb and venules (10%); disproportionate widening of efferent limb (6%)	It seems possible that the changes observed with the capillary microscope are the counterpart in vivo of the pathological lesions encountered on histological examination of the tissues.
Mc Gill et al., 1986	Observational, uncontrolled study	30 patients, 10 SSc, 9 SLE, 11 RA	9 of 11 RA patients had normal capillaries or minor non-specific abnormalities (very thin capillary loops), 2 patients had technically unsatisfactory results, none had enlarged capillaries.	Nailfold capillaroscopy is effective for the differential diagnosis between SLE, SSc and RA.
Drevet et al., 1986	Observational, uncontrolled study	80 RA patients	Excessive erythrocyte aggregation and/or pericapillary edema in 74% of patients, capillary neogenesis in 59%, spontaneous hemorrhage in 28% and stigmata of cutaneous atrophy in 54%.	Findings demonstrate the frequency of subclinical cutaneous damage in rheumatoid disease.
Altomonte et al., 1995	Observational, uncontrolled study	32 RA patients	Elongated, tiny and tortuous capillaries. Higher subpapilar venous plexus visibility in patients presenting antinuclear antibodies and antibodies against rheumatoid-arthritis-associated nuclear antigen (anti-RANA antibodies)	No differences in the capillaroscopic pattern were found between rheumatoid factor positive and rheumatoid factor negative patients, elongated and tortuous capillaries seem to be the main alterations in RA, however they are not specific to the disease and are not correlated with the presence of RF
Kuryliszyn-Moskal et al., 1996	Case-control study	79 RA patients and 30 healthy controls	75% with severe vascular changes exceeded normal sICAM-1 cut off value	No significant correlation between sICAM-1 levels and capillaroscopy findings
Kuryliszyn-Moskal et al., 1998	Observational, uncontrolled study	80 RA patients	Capillary abnormalities were observed in 75 RA patients (93.8%). Tortuous or meandering pattern was observed. Mild capillaroscopic changes found in 16.3%, moderate in 56.3% and severe in 21.2% of patients	A significant correlation between sCD4 levels and the capillaroscopy findings was found. The group with severe capillaroscopic changes had a longer disease duration, higher frequency of joint erosions and systemic involvement
Witkowska et al., 2003	Case-control study	37 RA patients and 18 healthy controls	higher sICAM-1 level appear to reflect more intensive capillaroscopic changes	Compared with controls, all RA patients had significantly lower serum selenium concentrations, irrespective of the degree of capillaroscopic vascular changes.
Scardina et al., 2006	Case-control study	30 RA patients and 30 healthy controls	Reduced capillary tortuosity and caliber, elongated capillaries	Capillaroscopy was useful in assessing the labial mucosa microcirculation
Dolijanović et al., 2006	Observational, uncontrolled study	250 children with signs of connective tissue disease (mean age 15 years)	At follow-up 10 (4%) patients had rheumatoid arthritis (6 of them juvenile onset rheumatoid arthritis) 7/10 patients had normal capillaroscopic findings 3/10 had non-specific findings	No specific capillary changes predictive for future development of juvenile onset RA
Lambova et al., 2012	Case-control study	62 RA patients and 62 healthy controls	Prominent subpapillary plexus (69%); elongated capillaries (58%); dilated capillaries (78.9% in RA patients with RP, 62.8% in RA patients without clinical symptoms of vasospasm of peripheral vessels; “scleroderma-like” pattern (14.5%)	The diagnostic use of nailfold capillaroscopy was confirmed. Capillaroscopy proved necessary during follow-up for RA patients with SSc pattern findings for the early detection of a systemic rheumatic disease, different from inflammatory arthritis
Lambova et al., 2012	Comparative study	93 patients with rheumatic disease	quantitative and qualitative assessment detected dilated and giant capillaries, avascular areas, haemorrhages	No significant difference between qualitative and quantitative methods of assessment was found for the detection of avascular areas. Quantitative analysis is more precise especially for the detection of capillary dilation.
Graceffa et al., 2013	Case-control study	30 RA patients, 30 PsA patients and 30 healthy controls	Significantly increased venous limb diameter, arterial limb diameter, loop diameter and loop amplitude in RA patients compared to controls (*p* < 0.001 for all comparisons)	Capillaroscopy can serve as a diagnostic tool for the differential diagnosis between PsA and RA. Microvascular structural abnormalities might reflect endothelial injury due to systemic inflammation Caused by chronic arthritis.
Errichetti et al., 2016	Case-control study	12 patients with RA, 15 PsA patients sine psoriasis, and 12 controls	Parallel dotted/short linear vessels (58.3%); irregular/ramified, blurry, purple vessels (33.3%); reddish background without distinct vessels (8.3%)	Capillaroscopy may be a useful supportive tool for differentiating earsy PsA sine psoriasis from RA
Sag et al., 2017	Case-control study	201 RA patients and 50 healthy controls	Non-specific capillaroscopic findings (45.77%); tortuosity (49.5%); microhaemorrhages (27.9%); dilated capillaries (17.9%); bushy capillaries (16.9%); ramified capillaries (10%); avascular areas (5%); capillary density <10 per 1 mm^2^ (4.5%)	there is a weak relationship between tortuosity and the duration of disease, no significant relation was detected between capillaryscopy findings and parameters such as RF, anti-CCP positivity and DAS28
Rajaei A et al., 2017	Observational, uncontrolled study	430 RA patients	Tortuosity (99.5%); angiogenesis (74.7%); scleroderma pattern (20.9%); normal pattern (7.2%)	NFC may be valuable for disease monitoring in RA as well as for patients’ follow-up
Anyfanti et al., 2018	Case-control study	99 RA patients and 35 controls	Decreased capillary density	Capillary rarefaction in RA correlates with lower cardic output, increased arterial stiffness and cardiovascular risk
Bernardino et al., 2019	Observational, uncontrolled study	22 RA patients (91% with RP) and 362 patients with other rheumatic diseases	Tortuosity (100%); crossing capillaries (100%); enlarged capillaries (68.2%); haemorrhages (59.1%); giant capillaries (13.6%); elongated capillaries (18.2%); scleroderma pattern (4.55%); neonagiogenesis (4.55%)	Capillaroscopic changes appear to have no correlation with disease activity. Dilated or giant capillaries require a closer follow-up.
Van Roon et al., 2019	Case-control study	759 patients (354 normal), 15 with RA	SSc pattern on NCM was observed in 13% of RA patients	SSc pattern is associated with high prevalence of abnormal PFT
Faruk Elmas et al., 2020	Case-control study	59 RA patients and 60 healthy subjects	Megacapillaries (43.1%); capillary deformity (41.2%); avascular areas (23.5%); capillary vascular anomalies (52.9%)	No correlation of findings with DAS28 score

RA: rheumatoid arthritis, RP: Raynaud’s phenomenon, PsA: psoriatic arthritis; SSc: systemic sclerosis; SLE: systemic lupus erythematosus.

## Data Availability

This review article does not report any primary data. It summarizes data reported in studies published previously.
